# Silk films with nanotopography and extracellular proteins enhance corneal epithelial wound healing

**DOI:** 10.1038/s41598-021-87658-1

**Published:** 2021-04-14

**Authors:** Yuncin Luo, Kai B. Kang, Rachel Sartaj, Michael G. Sun, Qiang Zhou, Victor H. Guaiquil, Mark I. Rosenblatt

**Affiliations:** Department of Ophthalmology and Visual Sciences, University of Illinois at Chicago, 1855 W. Taylor Street, MC648, Chicago, IL 60612 USA

**Keywords:** Biochemistry, Biological techniques, Biotechnology, Cell biology, Genetics, Medical research, Materials science, Nanoscience and technology, Optics and photonics

## Abstract

Corneal wound healing depends on extracellular matrix (ECM) and topographical cues that modulate migration and proliferation of regenerating cells. In our study, silk films with either flat or nanotopography patterned parallel ridge widths of 2000, 1000, 800 nm surfaces were combined with ECMs which include collagen type I (collagen I), fibronectin, laminin, and Poly-d-Lysine to accelerate corneal wound healing. Silk films with 800 nm ridge width provided better cell spreading and wound recovery than other size topographies. Coating 800 nm patterned silk films with collagen I proves to optimally further increased mouse and rabbit corneal epithelial cells growth and wound recovery. This enhanced cellular response correlated with redistribution and increase in size and total amount of focal adhesion. Transcriptomics and signaling pathway analysis suggested that silk topography regulates cell behaviors via actin nucleation ARP-WASP complex pathway, which regulate filopodia formation. This mechanism was further explored and inhibition of Cdc42, a key protein in this pathway, delayed wound healing and decreased the length, density, and alignment of filopodia. Inhibition of Cdc42 in vivo resulted in delayed re-epithelization of injured corneas. We conclude that silk film nanotopography in combination with collagen I constitutes a better substrate for corneal wound repair than either nanotopography or ECM alone.

## Introduction

Damage to the ocular surface can occur by traumatic, chemical, surgical or immune-conditions, resulting in injuries of the corneal epithelium, which requires mechanochemical signals to promote wound healing. In this regenerative process, the corneal epithelial basement membrane, a layered cell-adherent ECM, provides biochemical and topographic features to allow for epithelial cell attachment and migration. In addition to the well-documented biochemical signals that participate in corneal wound healing, the basement membrane surface provides important cues that act as mechanotransducers for cell growth^1–3^. Previous experiments using scanning electron microscopy (SEM) have demonstrated that the layer between corneal epithelium and stroma possesses a rich 3-dimensional nanoscale topography in the 50–200 nm range^4,5^ which provides a physical stimulus, that modulates cell-substratum adhesion. Furthermore, it has been shown that micro- and nanotopographical cues can influence cellular migration, adhesion, proliferation and differentiation in human corneal epithelial cells^6–8^. Since both biochemical and topographical cues can modulate cellular growth, attention to both types of signals when designing a biomaterial scaffold for corneal tissue repair might be considered.

Currently, there are different approaches for repairing damaged cornea including the use of cell transplantation, amniotic membrane, fibrin gels, polymers, and biopolymers^9^. Recently the use of solubilized silk in regenerative medicine as sponge scaffolds, hydrogels, and films, has made silk an attractive biomaterial for corneal repair^10,11^. Silk has been historically used as a biocompatible and nonimmunogenic biomaterial in surgical procedures. Properties such as its malleable shape, controllable degradability and mechanical strength make silk a biomaterial with strong potential for bioengineering purposes. Silk film surfaces are also easily manipulated with topographical feature by soft-photolithography technology and its highly optical transparency property makes it suitable for ophthalmological applications.

Previously we had shown that micro- and nanoscale patterned silk film substrates may serve as scaffolds that provide biophysical cues to epithelial progenitor cells^12–16^. In particular, we have studied human corneal limbal-epithelial (HCLE) cells response to flat silk and varied geometric topography in vitro. We found that cells were able to generate cell sheet on flat silk film^12^, cell behaviors response differed to flat, ringed, lined silk topographies. Ringed and lined topographies with 2000 nm width and 4000 nm pitch change cell–cell contact, had elongated cell morphology, rearranged cell cytoskeleton and focal adhesion proteins. These silk topographies increased initial adhesion and cell number compared to glass, flat and ring silk surfaces but not increased cell proliferation^13^. However, they induced cell sheet migration when compared to flat silk surfaces^14^. Our recent studies shown that human corneal epithelial cells grown on silk film nanotopographies, differentially expressed genes involved in actin organization, integrin signaling, and focal adhesion kinase signaling and maintain corneal epithelial stem cells at a less differentiated state^15,16^. From these previous findings, we hypothesized that silk topographies provide physical cues that may influence cellular adherence and migration by mechanisms related to cytoskeleton rearrangement.

It is well documented, that beside the effect of topographies, ECM modifications also impact cellular behavior and need to be considered for any scaffold design aiming at promoting corneal epithelium wound healing. In many species including humans, there is an extra membrane layer, called Bowman’s layer composed of collagen I^17^. This membrane lies between the corneal epithelium basement membrane, which is mainly composed of laminin, fibronectin, and collagen IV^18^, and the stroma. These ECM proteins have a profound role in the regeneration of wounded corneal epithelium. In this manuscript we have combined the use of silk films of different topographic ridge size and different ECM coating, such as collagen I, laminin, fibronectin or Poly-d-Lysine, to evaluate their effect on corneal epithelial cell behavior. Additionally, we explored the effect of these combinations on corneal epithelium wound healing, an aspect that has not been yet studied.

## Materials and methods

### Animals

All animal experiments were approved by the Institutional Animal Care and Use Committee from the University of Illinois-Chicago under the approved protocol 20-221, following the US NIH Guide for the Care and Use of Laboratory Animals and guidelines of the Association for Research in Vision and Ophthalmology Statement for the Use of Animals in Ophthalmic and Vision Research and in compliance with the Arrive guidelines. Wild‐type CD1 mice were obtained from Jackson Laboratories (Bar Harbor, ME) and New Zealand white rabbits from Charles River Laboratories International, Inc. (Wilmington, MA).

### Silk fibroin production

Silk fibroin was extracted from Bombyx mori silkworm cocoons (Tajima Shoji Co., Yokohama, Japan) as previously described^13–16,19–21^. In short, cocoons were weighed, cut, and boiled for 40 min in 0.02 M Na_2_CO_3_ (Sigma-Aldrich, Saint Louis, MO) to remove sericin protein. The fibroin fibers were rinsed three times in dH_2_O for 20 min per wash. The fibroin fibers were air dried overnight in a laminar flow hood. The next day, the dry fibroin fibers were dissolved in 9.3 M LiBr solution in a 60 °C water bath for 4 h. The LiBr was removed from silk fibroin by dialyzing in water in a MWCO 3,500 Slide-A-dialysis cassette (Thermo Fisher, Waltham, MA) for 48 h. After dialysis, the remaining cocoon debris were eliminated by centrifuging the dialyzed silk solution twice at 13,000*g* at 4 °C. The supernatant was collected and stored at 4 °C. The final concentration of aqueous silk solution was 8 wt./vol.%, as determined by gravimetric analysis.

### Patterned silicon wafer production

Silicon wafers with parallel ridge widths of 2000 nm, 1000 nm, and 800 nm at 1:1 ratio to groove and 1 μm depth were purchased from a photolithography service lab (LumArray Inc., Somerville, MA). The silicon wafers were produced by zone-plate-array lithography^22,23^ and ion etching techniques. These wafers were used as master molds to cast PDMS (Polydimethylsiloxane) that eventually were used to produce silk films with distinct topographies. Topography sizes were chosen according to previous cell-topography interaction studies. Our previous studies showed that ridge widths of 2000 nm, 1000 nm, and 800 nm have differential effects on cell morphology, cell elongation, and gene expression^15,16^.

### Silicon wafer surface silanization

Due to the tight adhesion between PDMS and silicon wafer molds, PDMS damages fine pattern on the silicon wafer molds when separate them by peeling. To protect and make the silicon wafer molds reusable we pre-treated silicon wafer surfaces with organofunctional alkoxysilane molecules before casting PDMS, this process is called silanization. For the silanization process, silicon wafers were placed inside a desiccator with an aluminum foil cup containing one drop of tridecafluoroctyltrichlorosilane (UCT specialties, LLC of Bristol, PA) by the side^24,25^. The vacuum desiccator was placed in a laminar hood overnight. By vapor-phase deposition, tridecafluoroctyltrichlorosilane formed an anti-stick monolayer on the silicon master mold surfaces.

### PDMS mold production

PDMS molds were made of an elastic polymer which is composed of two ingredients: the base and curing agent. Here we used the curing agent Sylgard 184 silicone elastomer kit (Dow Corning, Midland, MI) silicone and mixed the base and curing agent at a 10:1 ratio by weight. After mixing well, the PDMS mixture was degassed until all bubbles disappeared and then poured on the silicon wafers as previously described^26,27^. The casted PDMS was then allowed to polymerize in an oven at 60 °C overnight. The following day the cured PDMS were removed from the silicon substrate and then punched to form around 14 mm circles. The surfaces were cleaned with 70% ethanol, three distilled water rinses, and then allowed to air dry in a clean environment^24,28,29^.

### Silk film casting and sterilization

Silk films were produced by casting 75 μL of 8% silk fibroin solution onto the round 14 mm PDMS surfaces to produce silk films with 40 μm in thickness as previously described^30^. Silk films were allowed to dry overnight to form the patterned surfaces. Silk films on the top of PDMS molds were then water-annealed (WA) by placing the samples in a water-vapor-saturated desiccator for more than 4 h. This process induces β-sheet protein secondary structure formation and produces a water-insoluble silk films for cell culture. After the WA process, silk films were removed from their PDMS molds and hydrated with sterilized deionized water. Once the silk films became hydrated and soft, they were incubated in 70% ethanol for 10 min and washed with ddH_2_O 3 times. Silk films and glass cover slips were placed with their pattern side up into 24-well plates, and a stainless steel O-ring was placed on top to hold the films down to the bottom of the culture well. Films were then subsequently washed three times with 1 mL of sterile phosphate-buffered saline (PBS). The silk films were left in the final PBS wash until ready for ECM coating or cell seeding^31–33^.

### Coating of silk film with ECM

Silk films were coated with rat collagen I (Gibco, Thermo Fisher), fibronectin (BD Biosciences, Bedford, MA, USA), mouse laminin (Invitrogen, Thermo Fisher) or Poly-d-Lysine (Gibco, Thermo Fisher) respectively in a 50 μg/ml concentration diluted in dH_2_O. We used 200 μl ECM to coat the silk film surfaces and incubated for 2 h at room temperature (RT). Then washed with 1 ml PBS for 3 times. These coated silk films were prepared freshly before seeding cells^34–36^.

### Primary mouse and rabbit corneal epithelial cells isolation

Mouse corneal epithelial cells (MCEC) were isolated from 2–3 months old CD1 mice^37^. Mice were euthanized with CO_2_ followed by cervical dislocation. The enucleated eyes were washed 3 times in PBS containing 1% Penicillin–Streptomycin (P/S) (Corning Life Science, Corning, NY) and incubated at 4 °C for 18 h in CnT-Prime medium (CELLnTech, Bern, Switzerland) containing 12.6 unit/ml Dispase (Gibco, Thermo Fisher) and 100 mM D-Sorbitol (Sigma-Aldrich). Mouse corneal epithelium could easily be peeled off from the stroma using a spatula. After getting a sheet of corneal epithelium, single corneal epithelial cells were obtained by digesting in 0.05% trypsin/0.03% EDTA (Life Technologies, Carlsbad, CA) at 37 °C in a water bath for 10 min and pipetting every 3 min to obtain a single cell suspension. The enzymatic reaction was stopped by adding DMEM medium containing 10% fetal bovine serum (FBS) (Sigma-Aldrich), and washed with PBS to remove DMEM and FBS. Cells were centrifuged at 1000 rpm for 5 min to remove trypsin EDTA and resuspended in CnT-Prime medium. Cell density was adjusted to 2 × 10^5^ in CnT-Prime medium containing 1% P/S.

Rabbit corneal epithelial cells (RCEC) were isolated from limbus tissue explants^38–41^. Briefly, rabbit eyes were repeatedly washed by shaking the eyes in fresh PBS with 1% P/S. Dissected corneoscleral rings were obtained by removal of the iris, residual conjunctiva, endothelial layer, and trabecular meshwork. A 2 mm in diameter corneal trephine punch was used to get a 2 mm limbus corneal tissue followed by immersion in FBS. The anterior epithelium was placed directly upside down on 10 cm tissue culture dishes and incubated in FBS at 37 °C in 5% CO_2_. After 30 min, the explants adhered to the plates more tightly and one drop of RCEC medium was added to each explant to prevent dry out. The explants were thus cultured overnight in the incubator at 37 °C. RCEC medium ingredients included DMEM, supplemented with 1% MEM non-essential amino acids (Gibco, Thermo Fisher), 1% P/S, 400 ng/ml hydrocortisone (Sigma-Aldrich), 5 μg/ml human transferrin (Sigma-Aldrich), 2 mM L-glutamine (Corning Life Science), 5 μg/ml insulin (Sigma-Aldrich), 15% FBS, 10 μg/mL mouse epidermal growth factor (Invitrogen, Thermo Fisher), and antibiotic–antimycotic solution (Corning Life Science). The next day, 6 mL of complete RCEC culture medium was added and the medium was changed every 3 days until 80% cell confluence was achieved; usually by the 12th day. RCEC were then harvested for cell number measurement and wound healing experiments.

### Cell counting and analysis

MCEC were seeded at a concentration of 2X10^5^ on silk films with or without ECM coating in a 24-well cell culture plate. Cellular spreading and growth behavior were recorded by taking phase-contrast images on day 1, 3, and 5 using a 10X objective in a Living cell microscope system (Carl Zeiss GmbH, Jena, Germany). Unattached cells were washed after 3 days because typically MCEC adhere to surfaces slower than immortalized cell lines^42^. Cells were counted as an adherent and spreading cell if they were attached and did not have a circular shape^43,44^.

### Focal adhesion protein and filopodia immunofluorescence staining

MCEC were cultured on glass, flat, and 800 nm silk films with or without collagen I coating and analyzed at day 1, 3, and 5 in culture. Cells were fixed with 4% paraformaldehyde (PFA) for 15 min, then washed with PBS for 3 times and permeabilized with 0.4% Tween-20 in PBS for 5 min at RT. The non-specific binding surface was blocked with blocking solution (5% donkey serum and 2% bovine serum albumin in PBS) for 30 min at RT. Focal adhesion (FA) complex were marked by vinculin antibody. Cells were incubated with 1:600 dilution of anti-vinculin primary antibody (V9131, Sigma-Aldrich) for 1 h at RT followed by incubation with Oregon Green 488 goat anti-mouse secondary antibody (O11033, Invitrogen, Thermo Fisher) at dilution of 1:800 for 1hour. F-actin and nuclei were then stained by incubating cells with Alexa Fluor 568-conjugated Phalloidin (A12380, Invitrogen, Thermo Fisher) at a 1:100 dilution and with 4′,6‐diamidino‐2‐phenylindole (DAPI) (AnaSpec Inc., Fremont, CA) at a 1:10,000 dilution for 30 min and 5 min respectively. After wash with PBS to remove the unbound excess dye, samples were mounted in Vectashield mounting medium containing DAPI (Vector Laboratories, Inc., Burlingame, CA) and protected with a glass coverslip. To identify morphology of filopodia, F-actin and cell membrane were visualized by the following protocol. Cells were fixed with 4% PFA for 15 min and washed with PBS for 3 times then incubated with membrane lipid dye, PHK67 green fluorescent cell linker kit (Sigma-Aldrich) for 1 h and the unbound PHK dye was washed by PBS. Later on, cell membrane was permeabilized with 0.4% Tween-20 for 5 min and incubated with Alexa Fluor 568-conjugated Phalloidin at a dilution of 1:100 for 30 min.

### Image analysis and processing

Vinculin, F-actin, and cell membrane fluorescent images were taken using 63X objective on a Yokogawa spinning disc incorporated with Zeiss Z1 microscope, operated by Zen Zeiss Software (Carl Zeiss) to avoid non-specific silk film fluorescent background, which is caused by natural tendency of silk films to absorb dye. Z-stack images (10 layers) were captured at 0.25 μm slices using DAPI, GFP, and Texas Red filter channels. Additionally, a bright light channel was used to image the patterned silk films. The puncta and the area of vinculin-positive FA was measured by using ImageJ (NIH). Focal adhesion size for individual vinculin punctate size was defined to those between the range of 0.25–10 μm^2^. Focal adhesion area was defined as the percent of vinculin area over the total cell area, and focal adhesion density as the number of vinculin punctate in a unit area, these parameters were quantified as previously described^13^. Filopodia imaging analysis was performed according to the following parameters. We first defined filopodia as a finger-like protrusion with a width less than 0.5 μm and a length more than 0.5 μm^45,46^. The average filopodial length was analyzed by Zen software (Carl Zeiss) length measurement tool. To analyze the long filopodial population, we defined ‘long-filopodia’ as longer than 4 μm. We measured at least 100 filopodia for each experiment for each surface. The arc-chord ratio (tortuosity) was defined as the ratio of total curve length to the linear length of the wound edge as described previously^14^. Alignment of each filopodia was defined as the filopodia deviation from the silk patterns. The angle between the long axis of the filopodia and the pattern ridge axis were measured by Zen software angle measurement tool. Over 100 filopodia were measured for each pattern to get all length, proportion of long filopodia (> 4 μm) population, filopodia density, and the filopodia intersect angles with patterns.

### Scratch and wound healing assay

To study the effects of silk topography on corneal epithelial cells wound healing, we performed in vitro scratch assay on RCEC^47,48^. Cells were seeded at a concentration of 2 × 10^5^ cells/well on a 14 mm diameter flat and 800 nm ridge size silk films, with or without collagen I coating, and grown until 100% confluent monolayers. To generate a cell-free area perpendicular to patterns, we made a linear scratch in the middle of the silk films. To make the scratch we chose a painting tool with a soft silicone tip, which made a linear wound on collective cell layer without damaging the underneath nanotopography, unlike plastic pipette tips which usually cause damage to the nanotopography. Cultures were gently washed 3 times with PBS to remove detached cells. The phase-contrast images were taken with the 10X objective every 20 min for recording the wound recovery process on a Live-Cell imaging microscope (Carl Zeiss) which has a 5% CO_2_ supply and 37 °C incubating humidity chamber culture system. We recorded 3 different fields per scratch on a silk film and performed triplicate experiments. Wounded areas were measured at time point 0-, 3-, 5-, 7-, 10- and 15- hours post scratch. Each wounded area was analyzed using a Zen software. Wound recoveries at each time point were shown as a percentage of the initial wound area. For filopodia analysis, cells were incubated for 4 to 7 h after scratch and imaged before the 100% recovery.

### Pathways analysis

Gene expression data obtained previously^15,16^, was evaluated for Ingenuity Pathway Analysis (IPA) (Ingenuity System Inc., Redwood City, CA). Briefly, human corneal epithelial cells (HCEC) were cultured on silk films with flat, 2000, 1000, and 800 nm ridge width topography. After 72 h in culture, cells were collected and total RNA obtained and used to construct a cDNA library by using TruSeq mRNA-seq Sample Preparation Kit (Illumina, San Diego, CA, USA). Illumina Hiseq 2500 sequencing system (Illumina, Inc., San Diego, CA, USA) was used for sequencing. Alignment of RNA-Seq reads against the human reference genome, were performed on SeqMan NGen assembler (DNAstar version 2016, Madison, WI, USA). The assembly was analyzed by DNAstar Qseq to generate normalized expression values of the transcript isoforms. Fold change between samples collected from different experimental conditions and the P value for each transcript were analyzed by ANOVA. Data set containing differentially expressed gene identifier and corresponding expression values were uploaded to Ingenuity Pathways Analysis. IPA uses the global molecular network developed in Ingenuity Pathways Knowledge Base (IPKB) which contains the biological function, interaction, and related biochemical pathways.

### Cdc42 inhibitor ML141 for in vitro and in vivo wound healing assay

Our IPA analysis demonstrated that silk topographies induce intracellular signals that regulates dynamic actin rearrangements and formation of lamellipodia and filopodia. A parallel pathway may start from integrin-binding and activation of Cdc42, which can also be induced by Rac signaling, leading to the formation of an ARP2/3 complex which induces actin nucleation and the formation of filopodia. There are limited identified inhibitors that specifically interact with small Rho family GTPases. We selected ML141, which is a cell permeable, allosteric, trisubstituted dihydropyrazolyl compound that has been demonstrated to be a potent, selective, reversible and non-competitive inhibit of Cdc42. This inhibitor has been shown to efficiently block Cdc42 association with GTPγS, decrease GTP-Cdc42 (> 95%), and inhibited the Cdc42-related filopodia formation^49,50^. For in vitro wound healing, ML141 (Selleckchem, Huston, TX) dissolved in DMSO, was added to the medium right before starting the scratch assay at a concentration of 50 μM and was kept in culture during the wound healing process. For in vivo wound healing, the effect of ML141 on corneal epithelium regeneration was tested on 12 weeks old C57BL/6 male and female mice. Mice were subjected to corneal epithelial debridement in one eye only as described^51^. In short, a 2 mm central corneal epithelium debridement was performed using an Algerbrush burr, then the injured area was delineated using 1% fluorescein and the eyes washes with PBS before imaging by a slit lamp. Immediately after imaging, mice received a single subconjunctival injection (5 μl) of 250 μM ML141 or 15 μl eyedrops of 500 μM ML141. A control group received 15 μl of vehicle (DMSO/PBS) as eyedrops. Eyedrops were applied at the time of injury and at 4, 8, 12, 24, 28 and 32 h post injury. Re-epithelization was followed up by slit lamp until full recovery. Images were quantified as described^51^.

### Statistical analysis

Statistical analysis was performed using Prism software (GraphPad, San Diego, USA). Student's t-test and Two-Way ANOVA with Tukey’s multiple comparison test were used for the difference. Data was considered significant different when P value is less than 0.05.

## Results

### Surface topography on silk film influences cornea epithelial cell number

**Figure 1:**
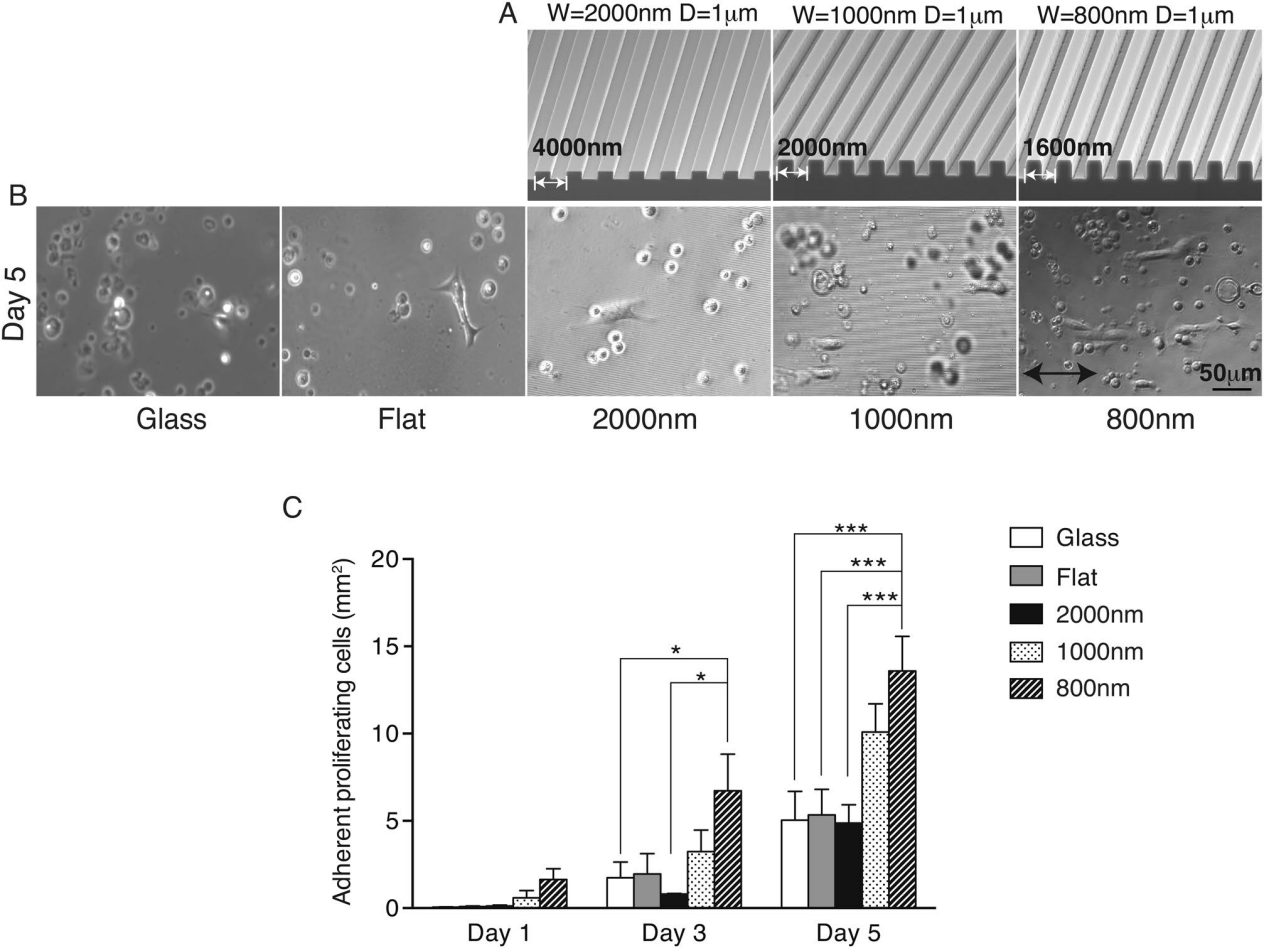
Nanotopography on silk films induced cell spreading and growth. (**A**) SEM images of silicon wafers with nanotopography patterns. The measurements of those ridge width sizes were 2000 nm, 1000 nm, and 800 nm and 1 µm in depth. (**B**) MCEC were seeded on a glass surfaces and 4 different silk topographies, cellular spreading and growth were followed up to 5 days. (**C**) Total cell number was analyzed by counting phase-contrast images taken at 1-, 3- and 5-day time points. (* indicates *p* < 0.05, ** indicates *p* < 0.005. Values are means ± S.E.M. The arrow indicates the direction of pattern axis).

The effects of silk film surfaces with linear topographical patterns of different ridge size on epithelial cells were studied using primary cultures of MCEC. Cells were allowed to attach and grow on various substrates, including glass coverslip as a control and silk films with flat surfaces and with different sized parallel line patterns as shown in Fig. 1A. The majority of MCEC were round on glass or flat silk film surfaces, while on the silk topographies the cells were elongated and aligned parallel to the direction of the patterned features (Fig. 1B). This effect was more pronounced as the cells were cultured for longer period of time as seen on day 5 of culture (Fig. 1C). In general, there were significantly more initial adhered cells on 800 nm and 1000 nm ridge width topographies at all time point. Spreading cell number on 800 nm pattern was 50-fold greater than on glass, 20-fold greater than on flat silk films, and twofold greater than on 2000 nm ridge width films at the 1-day time point; twofold more than on 1000 nm and threefold more than on 2000 nm patterned, flat silk films, and glass surfaces at 3-day time point; 2.5-fold more than other surfaces except 1000 nm feature significantly at 5-day time point.

### Coating silk films with collagen I significantly increases cell number while fibronectin and laminin have no effect

**Figure 2:**
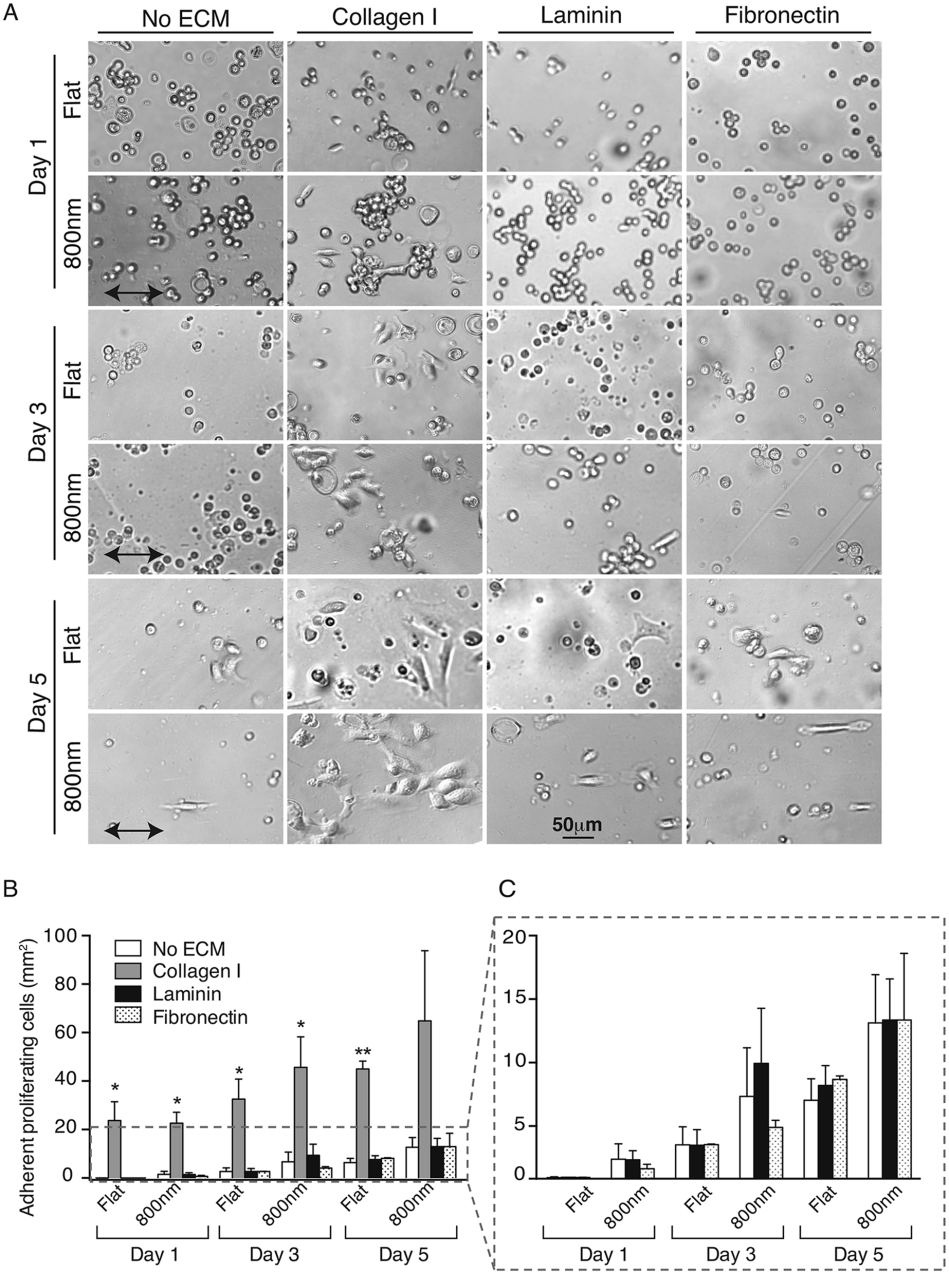
Collagen I coating of silk films enhanced cellular spreading and growth. MCEC were seeded on uncoated or collagen I, laminin, fibronectin or Poly-d-Lysine coated flat or 800 nm patterned silk films. (**A**) Representative phase-contrast images showing cellular shape and spreading. Higher cell number was observed on collagen I coated films through the days of analysis and the cell morphology and attachment was strikingly different to the effects of other ECMs. (**B**) Quantification of cell number showed that fivefold more spreading cells were present on collagen I coated silk in comparison to other ECMs. However, not significant differences were found between collagen coated nanotopographies and flat silk. (**C**) Detailed analysis of other ECMs, showed higher cell number on 800 nm patterned silk coated with laminin and fibronectin at 3-day time point when compared to uncoated flat silk or Poly-d-Lysine coating (data not shown). (* indicates *p* < 0.05, ** indicates *p* < 0.005 when comparing between ECMs. Values are means ± S.E.M. The arrow indicates the direction of pattern axis).

The aforementioned results suggested that silk films with 800 nm patterned topography had the greatest numbers of spreading cells and proliferation. Since ECM plays a major role on cellular behavior, we compared cellular growth on 800 nm and flat silk films coated with different ECMs that included collagen I, laminin, fibronectin, and Poly-d-Lysine. There were almost no MCECs adhered to Poly-d-Lysine coated substrates (data not shown). Quantification of the cell number (Fig. 2B) showed that cells grown on collagen I coated surfaces were significantly higher than those grown on films coated with other ECMs. Collagen I coating increased cell number 7–270-fold on flat and 5–13-fold on 800 nm silk films during 5 days in culture. Fibronectin and laminin coated silk films showed similar cell number compared to uncoated surfaces (Fig. 2C). Additionally, cells aligned with 800 nm ridge pattern on silk films only at 1-day time point on the collagen I coated silk films, thereafter the cell morphology became more amorphous at 3- and 5-day time point (Fig. 2A). However, most MCEC aligned with the direction of pattern when grown on fibronectin and laminin coated films.

Additionally, RCEC were used to determine if topography could influence the spreading and alignment of a second corneal epithelial cell type (Supplementary Fig. S1). Addition of collagen I significantly increased the cell number among all coated topographies (data not shown), with no significant differences between flat and 800 nm feature topography. Collagen I significantly increased cell number over other ECMs (Supplementary Fig. S1A). All other ECMs had variable effect on cell number, fibronectin had higher cell number only on flat films but not on the 800 nm topography, while laminin and Poly-d-Lysine did not have major effects (Supplementary Fig. S1B).

### Combination of collagen I coating and 800 nm patterned silk films provides better cell growth than each treatment alone

**Figure 3:**
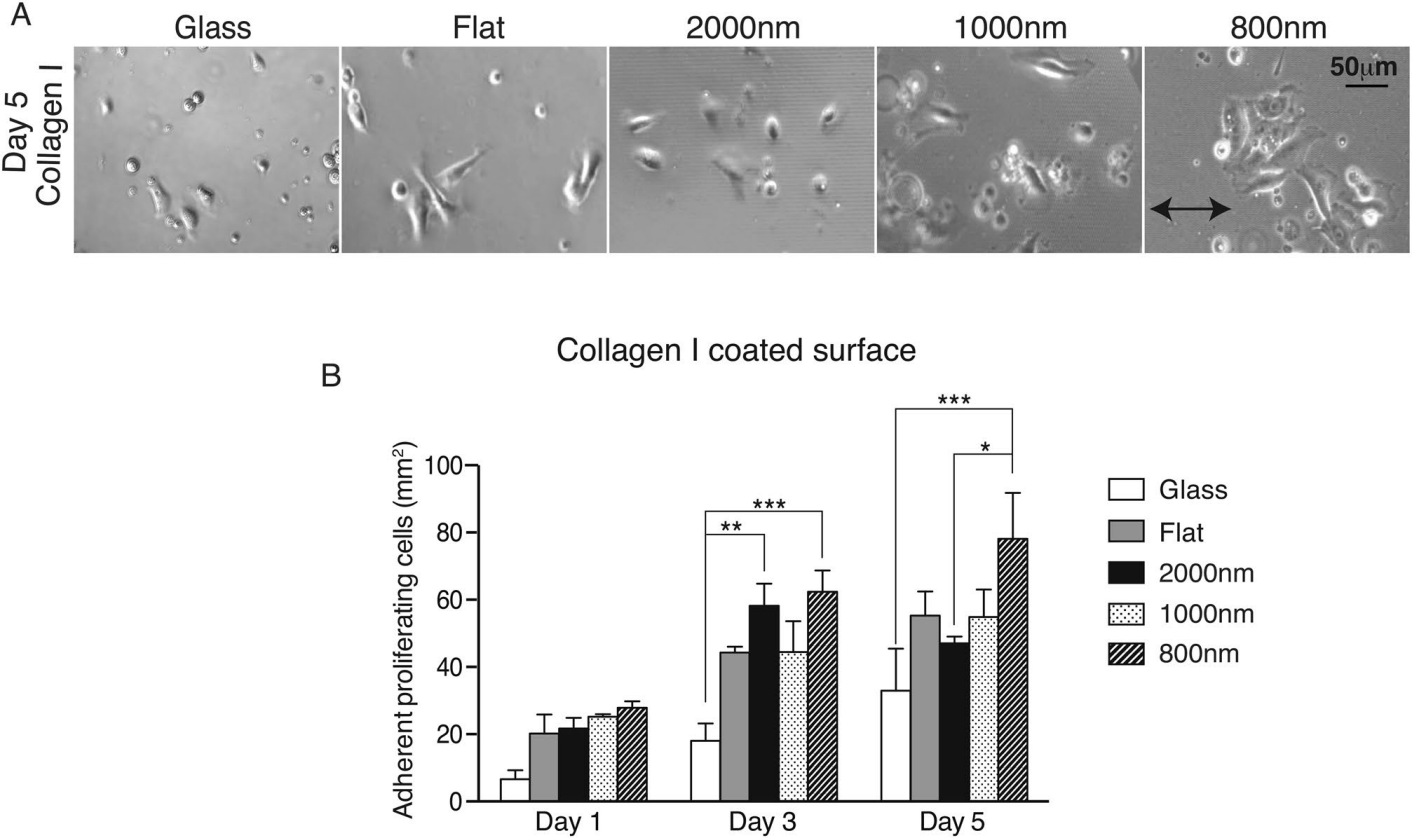
Combination of collagen I and smaller topography size resulted in improving cellular spreading and growth. (**A**) MCEC were seeded on collagen I coated glass and 4 different silk topographies; flat, 2000 nm, 1000 nm and 800 nm ridge width pattern. Phase-contrast images were taken at 5-day time point. (**B**) Quantification of cell number demonstrated that collagen I coating improved cell spreading and growth when compared to previous experiments. Silk films were better than glass and the smallest ridge width 800 nm showed the highest cell number (* indicates *p* < 0.05, ** indicates *p* < 0.005, ***indicates *p* < 0.0005. Values are means ± S.E.M. The arrow indicates the direction of pattern axis).

Since we found that collagen I was better than other ECMs at increasing cell number, we tested if this effect is dependent on the ridge width sizes of patterned silk films. We coated glass surfaces, flat silk, 2000 nm, 1000 nm and 800 nm patterned silk films with collagen I and analyzed the cellular growth at 1-, 3- and 5- day time points. Phase-contrast images showed that cell morphologies were amorphous and not aligned with the parallel line topography (Fig. 3A). Collagen I coating not only increased cell number on 800 nm patterns but also on all ridge sizes at all time points (Fig. 3B). At 1-day time point, there were 6 cells/mm^2^ on collagen I coated glass and 20–27 cells/mm^2^ on all collagen I coated silk films; at 3-day time point, there were 18 cells/mm^2^ on collagen I coated glass and 44–62 cells/mm^2^ on all collagen I coated silk films. At 5-day time point, there were significantly more cells on collagen I coated 800 nm silk films than on other surface. There were 78 cells/mm^2^ on 800 nm patterned silk films and 32–55 cells/mm^2^ on other surfaces. This experiment confirms our previous analysis and demonstrated that small silk nanotopographies combined with collagen I is better for cellular growth than combination of collagen I with bigger ridge width sized silk films.

### Focal cellular adhesion changes on silk films coated with collagen I

**Figure 4:**
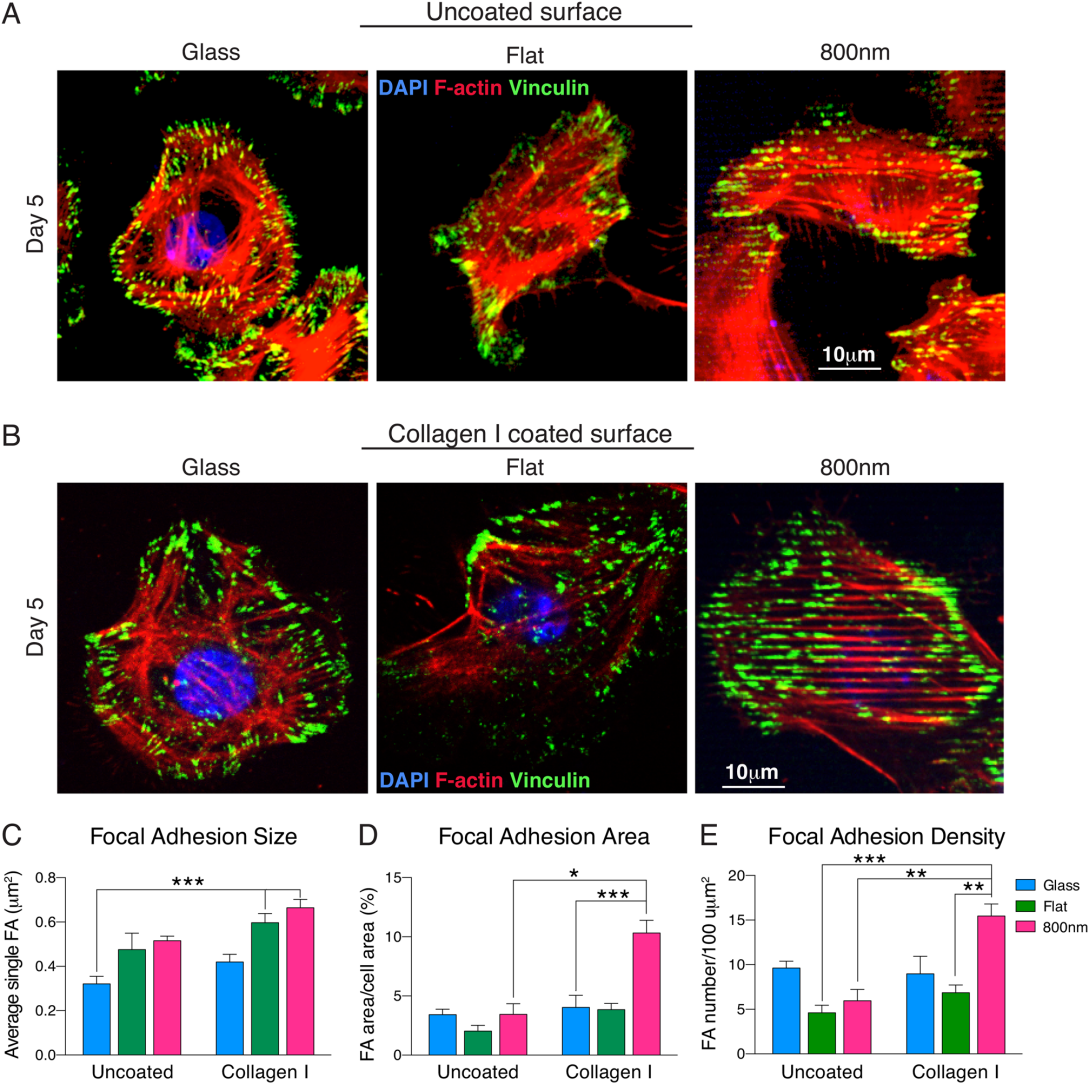
Smaller topography combined with collagen I coating induced higher cellular FA. MCEC were seeded on uncoated (**A**) or collagen I coated (**B**) glass, flat, or 800 nm silk films. After 5 days in culture, cells were stained for nuclei (blue-DAPI), vinculin (green- Oregon Green 488) and actin (red- Alexa Fluor 568) expression. Each vinculin punctate areas were analyzed to determine the FA size, FA area and density. (**C**) Individual FA size was defined by discrete areas of vinculin measurements and it was significantly larger on silk surfaces and addition of collagen I increased the cell FA size on each substrate. (**D**) The effect of collagen I was significantly evident when vinculin FA area was calculated, no major differences were observed on uncoated surfaces, but its addition to the 800 nm patterned silk films greatly increased the vinculin image area. (**E**) FA density was higher in cell cultured on glass across all uncoated substrate. However, addition of collagen I changed the vinculin density, especially on the 800 nm topography (* indicates *p* < 0.05, ** indicates *p* < 0.005, ***indicates *p* < 0.0005. Values are means ± S.E.M.).

We have shown that silk topography influences corneal epithelial cell FA localization^13^. The combinatorial effect of collagen I coating and surface topography was further investigated by analyzing the localization of vinculin, a focal adhesion protein, in MCEC seeded on glass, flat, and 800 nm patterned silk films. Cells were incubated for 5 days on uncoated or collagen I coated surfaces and analyzed by immunoflurescence staining. Cells grown on uncoated surfaces had mostly peripheral expression of vinculin (Fig. 4A), whereas those grown on coated surfaces showed less peripheral distribution but higher number of punctate vinculin (Fig. 4B). On collagen I coated or uncoated 800 nm patterned silk surface, vinculin punctate, as well as the actin fibrils, aligned with the pattern arrays. FA sizes on collagen I coated surfaces were bigger than uncoated surfaces (Fig. 4C). Collagen I coating showed minimal increase of FA area and FA density on glass and flat silk film but significantly increased in 800 nm patterned silk films: over threefold increase in FA area and twofold in FA density (Fig. 4D, E).

### Smaller ridge width topography and collagen I coating induce faster RCEC wound healing

**Figure 5:**
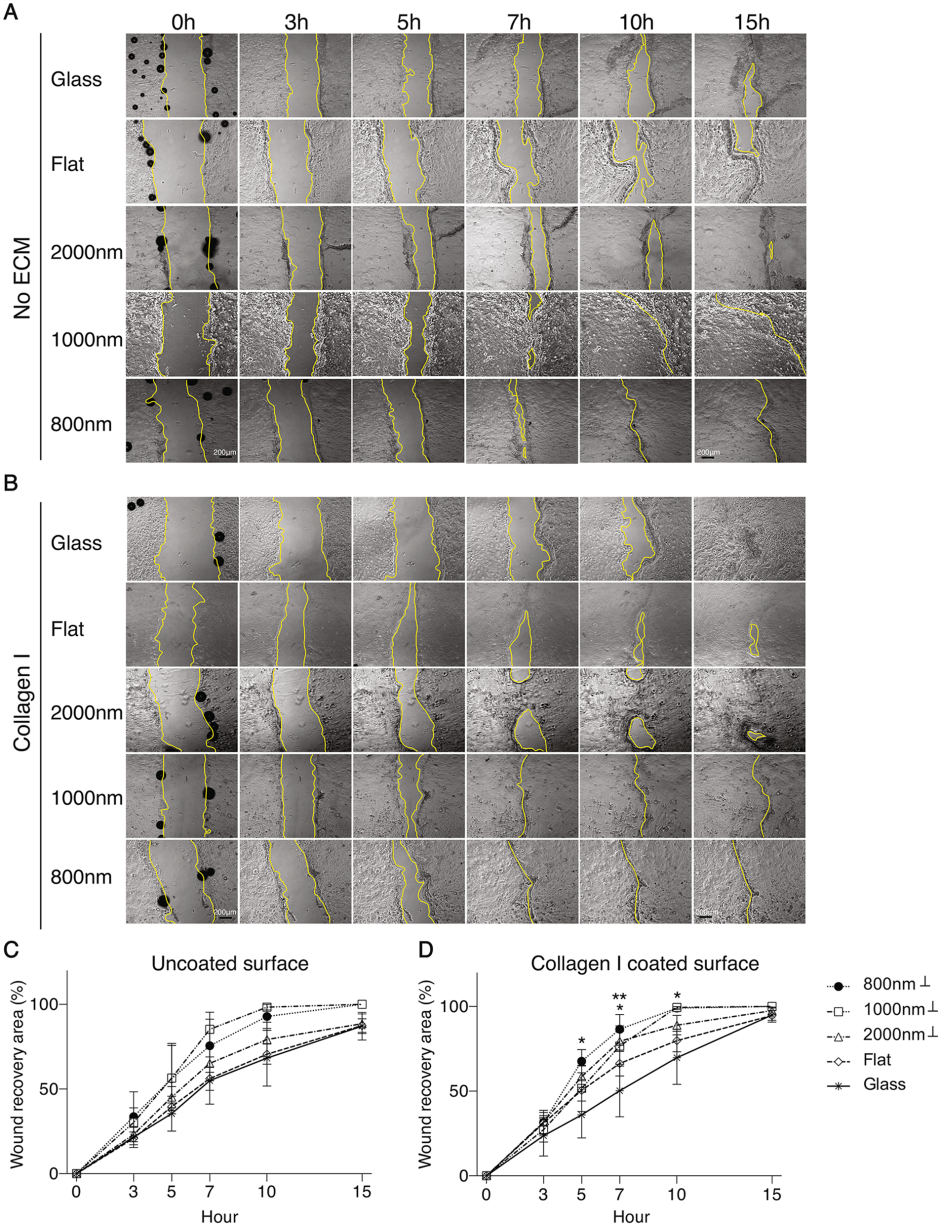
Nanotopographies and collagen I coating promoted in vitro wound healing of RCEC. Scratch assays were performed on confluent monolayer of cells grown on uncoated or collagen I coated glass, flat, 2000 nm, 1000 nm and 800 nm patterned silk films. Time-lapse images recorded every 20 min for 15 h were analyzed and the yellow lines delineated the wound edge of the scratched monolayer. Representative images of cells grown on uncoated (**A**) or collagen I coated (**B**) surfaces. Quantification of wound healing rate on uncoated surfaces shows that faster wound recovery occurred on the 1000 and 800 nm topography (**C**) and increased on the collagen I coated surfaces (**D**) (* indicates *p* < 0.05 when compared glass to 800 nm topography at 5-h time point; when compared glass to 2000 nm topography at 7-h time point; when compared glass to 800 nm topography at 10-h time point. ** indicates *p* < 0.005 when compared glass to 800 nm topography at 7-h tine point. Symbol on legends, ⊥, indicates the scratch was perpendicular to the parallel lines. Values are means ± S.E.M).

The potential effect of silk topography and collagen I coating on corneal epithelial cells wound recovery was determined using in vitro scratch assay of cultured primary RCEC seeded on different silk surfaces with collagen I coating (Fig. 5B) or without (Fig. 5A). We examined time-lapse images and evaluated the rate of wound closure by measuring the recovery area and expressed it as percentage of healing. On uncoated surfaces, we observed that the first wound to close occurred at 7 h post scratching on the 1000 nm patterned silk films followed by cells on 800 nm patterned silk films, while the last to heal were those seeded on glass surface (Fig. 5A). In collagen I coated silk films surfaces, cells seeded on silk films with small ridge topography (1000 nm and 800 nm) recovered faster than other surfaces (Fig. 5B). The quantification analysis showed that the recovery rate from fast to slow occurs in the following order: 1000 nm, 800 nm, 2000 nm, flat, and glass on both uncoated and collagen I coated surfaces and it was always faster on collagen I coated surfaces (Fig. 5C, D). On uncoated surface, wound recovery was faster on 1000 nm and 800 nm, 19% and 13% faster respectively, when compared to 2000 nm surface. However, wound recovery on 2000 nm patterned silk film was 7% faster than flat silk film and glass. The statistical analysis showed that the recovery speed increased on all surfaces coated with collagen I (3.8% on glass, 11.8% on flat, 10.6% on 2000 nm and 5.4% on 800 nm patterned silk films), but no significant increase was observed on 1000 nm patterned silk film (Fig. 5D).

### Ingenuity pathway analysis indicates that patterned silk films change the expression of genes involved in cellular cytoskeleton assembly

To determine the mechanism by which surface topography influenced wound recovery, we analyzed data from our previous study in which an RNA-sequence analysis was performed in HCEC grown on different silk film topographies^15,16^. The IPA analysis showed that differentially expressed genes were significantly linked to several canonical pathways which included Actin nucleation by ARP-WASP complex and Rac signaling (Supplementary Fig. S2A). From the top 14 pathways, the actin nucleation by ARP-WASP complex pathway was selected as a candidate for their role on cell adhesion, migration, and proliferation and because it becomes activated by mechanotransduction signaling. The analysis suggested that topography may regulate cell adhesion and wound recovery by effecting actin polymerization through this pathway, that includes proteins such as integrin, Cdc42, WAVE, N-WASP, and ARP2/3 (Supplementary Fig. S2B). The IPA canonical pathway also indicated that Rac/Rho signaling pathway were also upregulated on the 800 nm patterned topographies. The analysis indicates that the signal may start when the cells sense biophysical cues or ECM ligands provided by nanotopography on silk or collagen I through integrin receptors, which can aggregate and recruit a clustering of Rho family of small GTPases, which includes Rho and Rac. This will initiate a cascade of signaling events that globally regulates dynamic actin rearrangements. The signaling cascade may include activation of ROCK and MYPT leading to the formation of contractile actin filaments and formation of lamellipodia. A parallel pathway may start from integrin-binding and activation of Cdc42, which can also be induced by Rac signaling, leading to the formation of an ARP2/3 complex which induces actin nucleation and the formation of filopodia.

### Inhibition of Cdc42 delayed RCEC wound healing

**Figure 6:**
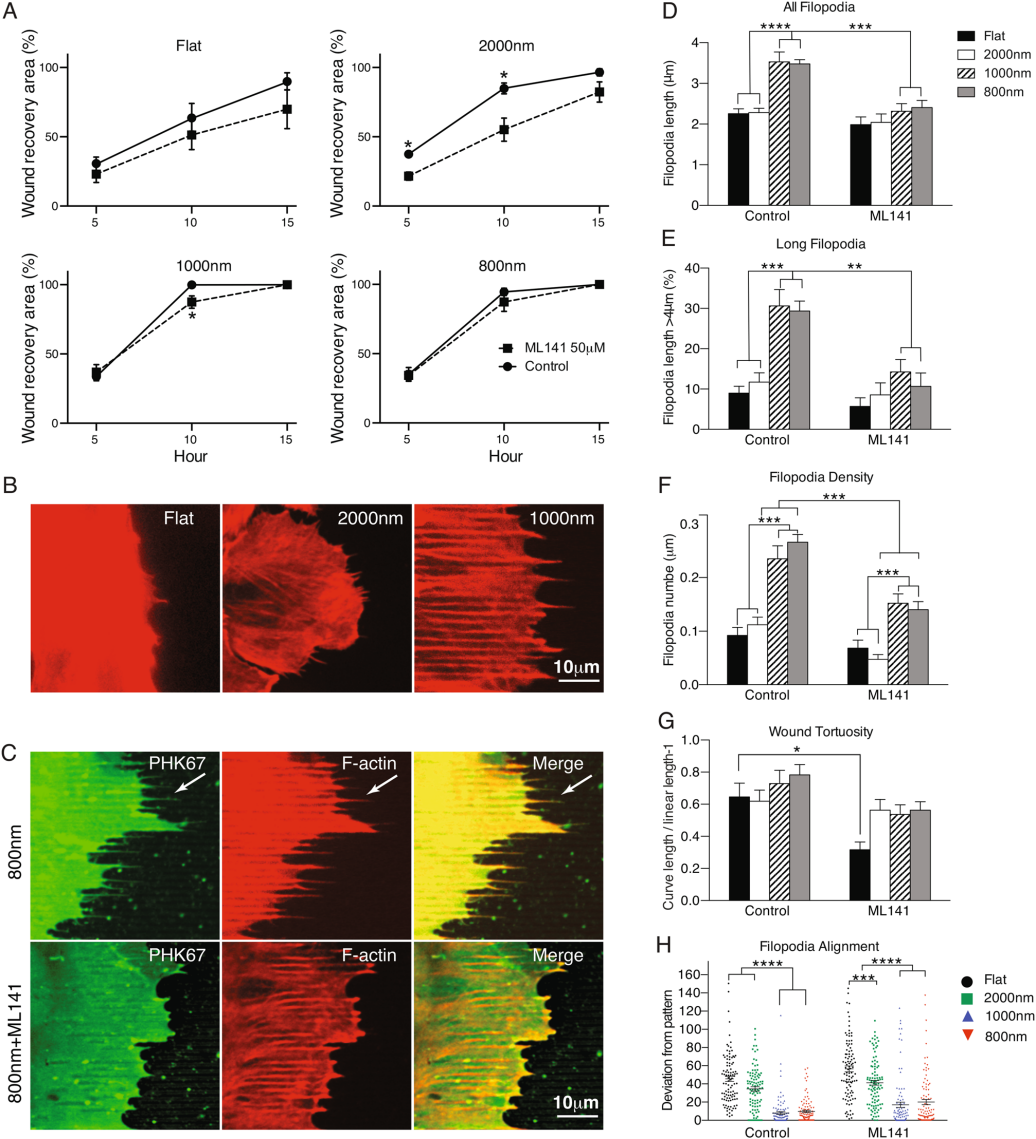
Filopodia formation is required for wound recovery of corneal epithelial cells. (**A**) Analysis of wound recovery indicated that the Cdc42 inhibitor induced delayed wound healing in all topographies but were significantly decreased on flat and 2000 nm patterned silk films (* indicate *p* < 0.05, when compared a time point between untreated and ML141 treated group. Values are means ± S.E.M.). (**B**) Filopodia was visualized by labeling F-actin with Phalloidin Alexa 568. Filopodia was clearly extended and aligned with the topography of 800 nm silk films. (**C**) Comparison of F-actin and membrane labeling of filopodia (PHK67) of cells on 800 nm silk films with or without ML141 treatment. (**D**–**H**) More than 100 filopodia were quantified and analyzed for extension of filopodia length, percentage of long filopodia population, and filopodia density. They were higher on silk films with smaller topography and always decreased upon inhibitor treatment. (**G**) The tortuosity analysis of wound edge showed that small topography induced more lamellipodia spreading than 2000 nm and flat silk films. (**H**) Filopodia alignment analysis showed that filopodia were more aligned with small topography than 2000 nm and flat silk films. After ML141 treatment, the tortuosity and alignment were reduced (N = 100). Angles between pattern axis and filopodia were measured and plotted (* indicates *p* < 0.05, ** indicates *p* < 0.005, ***indicates *p* < 0.0005 when compared between groups and between treatments. Values are means ± S.E.M.).

Our IPA analysis indicated that surface topography may influence cell migration through Cdc42, a central mediator that induces actin polymerization and the formation of filopodia (see Fig. S2). To validate this finding, ML141, a specific Cdc42 inhibitor was used to test the in vitro and in vivo effects of inhibit this signaling pathway. In vitro, we evaluated cell migration and filopodia formation in corneal epithelial cells subjected to scratch assays. RCEC were seeded on four topographies with or without 50 μM ML141 treatment and the wound recovery area, filopodia formation and distribution were quantified on time-lapse images (Fig. 6). In general, addition of the inhibitor decreased wound healing on all topographies but was clearly evident on flat and 2000 nm silk films. The average reduction (average of all time points analyzed) in wound recovery was 17.50% on flat, 29.0% on 2000 nm, 3.07% on 1000 nm and 2.66% on 800 nm (Fig. 6A). In vivo, mice were subjected to corneal epithelium debridement and treated with ML141 as eyedrops or subconjunctival injection and the re-epithelization of the injured cornea was followed up by slit lamp. We found that ML141 had a profound effect in the wound healing by delaying the re-epithelization. The effect were more severe when applied as eyedrops and this group of mice healed 3.5 days after the final addition of eyedrop treatment (Supplementary Fig. S4).

### Filopodia formation is induced by small topographies and decreased by inhibition of Cdc42

Actin filament structures are generally found in the leading edge of cells under migration and the formation of filopodia is essential for cell sheet migration especially in tissues such as the cornea where cells move from the limbus to the center of the eye. Since filopodia formation requires activation of Cdc42, we tested its role on wound healing. RCEC were seeded on different silk topographies and subjected to scratch assay when reached confluency. We observed distinct morphology of lamellipodia or filopodia in the leading edge of cells on patterned silk films and flat silk films. A lamellipodia is a thin (0.1–0.2 μm length) sheet-like protrusion that is filled with a branched network of actin. By contrast, filopodia are thin (0.1–0.3 μm length), finger-like structures that are filled with tight parallel bundles of filamentous (F)-actin^46^. In silk films with topographies, there were more finger-like structures stretching out on the edge of wound healing and in the direction of the pattern (Supplementary Fig. S3). F-actin and filopodia membrane staining showed that longer filopodia are formed on the 1000 nm and 800 nm topographies when compared to on flat silk or on 2000 nm patterned silk films and the filopodia aligned with the silk pattern (Fig. 6B, C). The average of all filopodia length was 2.2 µm on flat and 2000 nm, while it was 3.5 µm on 800 nm and 1000 nm patterned silk films (153% longer) (Fig. 6D). When cells were treated with the Cdc42 inhibitor ML141, filopodia shrunk among all surfaces (images not shown), especially on the 800 nm and 1000 nm patterned topographies, the average length decreased from 3 µm to 2 µm (Fig. 6C, D). The population of filopodia with length longer than 4um was 8.98% on flat, 11.73% on 2000 nm, 30.62% on 1000 nm, and 29.34% on 800 nm patterned silk films (Fig. 6E). After ML141 treatment, the population of filopodia with length longer than 4 µm decreased to an average of 5.67% on flat and 8.54% on 2000 nm but it was drastically reduced to 14.63% and 10.64% on 1000 nm and 800 nm patterned silk films respectively. Filopodia density was twofold more on 800 nm and 1000 nm than on flat and 2000 nm patterned silk films and decreased to a half after ML141 treatment (Fig. 6F). Wound tortuosity, which indicates how spread the wound healing is in the scratch assay, showed not significant different among surfaces but ML141 treatment made the wound leading surface less tortuous among all surface and significantly decreased on flat silk film surfaces (Fig. 6G). The angle of filopodia deviating from the pattern axis indicates how the filopodia aligns to the topography, the mean deviated angle from the pattern axis was only 7°–9° on 800 nm and 1000 nm while it was 45°–34° deviation on flat and 2000 nm (Fig. 6H). After ML141 treating, the filopodia deviated angle from pattern enlarged on 800 nm and 1000 nm topographies. However, the significant angle deviation difference among all topographies still existed after ML141 treatment (Fig. 6H).

## Discussion

We have previously shown that nanotopography on silk films induced changes in gene expression, cellular adherence, cytoskeleton rearrangement, collective cell sheet migration, and cell proliferation^12–16,19,33,52^. However, the surface topography features of silk have not been combined with ECM proteins that provide additional signaling inputs to drive cell growth. In this study, we have used silk with different topographic ridge size and combined with ECM such as collagen I, laminin, fibronectin or Poly-d-Lysine, to evaluate their effect on corneal epithelial cell behavior and wound healing. We found a clear effect of silk topography on MCEC, which do not adhere to glass or flat silk film surfaces, but the spreading cell number increased up to 50-fold on 800 nm patterned silk films. We reported a 36–50% increase cell number of HCLE seeded on microscale patterned silk films (2000 nm) when compared to ringed or flat silk films^13^. In this study we found that nanoscale topography (800 nm) had more MCEC than microscale (2000 nm) topography. We have shown that cells prefer to move parallel to the topography and observed increased collective cell sheet elongation and movement in the 2000 nm ridge width topography when compared to flat silk^14^. In this study, we further demonstrated that not only the direction of patterns but the nano-ridges size facilitated wound recovery. We clearly observed a correlation between increased FA and increased cell number in our study. Uncoated 800 nm silk films have significantly more cells, up to twofold more than other larger surface topographies, and correlated with a twofold increase in FA as well, suggesting that a smaller nanotopography provided more frequent periodic biophysical cues and more surface area for cells to form organized and bigger FA resulting in stronger cell attachment^53^. Interestingly, nanotopography did not change the amount of FA but influenced the distribution and density.

When we combined silk film topography with ECMs, we found that addition of collagen I increased cell number and enhanced FA distribution (FA size, total FA distribution, and FA density) on all surfaces, especially in the 800 nm, which also had one third more cell number. The synergistic effects of collagen I and 800 nm topography begins to appear on days 3 and 5 of culture. Collagen I coating provided a biochemical signal to the topographical effects induced by the tuned surfaces. Adding a peptide motif for cell receptor-ligand binding on an organized topography leads to further cell-substrate interaction^34,54^. Cell number on collagen I coated 800 nm was greater than the sum of collagen I coated flat silk films and 800 nm patterned silk films respectively. The combined effect was more than their individual augmentative effect, suggesting that the two properties worked in synergy. MCEC growth on collagen I coated 800 nm silk films had 2.5-fold more total FA area when compared to collagen I coated flat or uncoated 800 nm patterned silk films. This increase in vinculin expression (FA area; vinculin percent image area) could explain the synergistic effect observed. Besides, MCEC aligned with the pattern axis of the topography, but they did not when collagen I was present. This may be due to the presence of ligands covering the silk pattern both ridge and interval spacing, providing more desirable supporting points for cellular binding. Additionally, we found that fibronectin or laminin did not result in increased cell number which is in agreement with other studies showing that freshly isolated corneal epithelial cells spread on collagen I and IV, but not on fibronectin and laminin^55^. In a normal tissue, the corneal Bowman’s layer is a collagen I rich environment and contain less fibronectin and laminin^17^, and the corneal epithelium basement membrane contains several other collagens^18,56^, then it makes sense that cells adhere and proliferate better in collagen coated surfaces.

We proposed that cell surface integrin receptors sense biophysical cues provided by topographical patterns on silk and this signal are enhanced by the presence of collagen I. The clustering and activation of integrin receptors can activate Rho/Rac/Cdc42, members of rho- and ras-related superfamily of small GTPases, assemble focal adhesion kinase and recruit other FA protein, such as vinculin. Two related pathways can be activated, one from Rho-induced focal adhesion complexes which regulate the formation of stress fiber, and the other from Cdc42 and Rac which induce plasma membrane focal adhesion complexes and trigger the cortex actin polymerization, resulting in filopodia and lamellipodia formation^57^. Cdc42 effect on actin remodeling regulates filopodia formation and is in part mediated by members of a relatively well-studied family of proteins, the Wiskott Aldrich Syndrome protein (WASP) family^58,59^, which are ubiquitously expressed and connect Cdc42 to an actin nucleator, the Arp2/3 complex. This hypothesis was based on our IPA analysis of HCEC grown on different silk topographies which indicated that 14 top canonical pathways involved in cell adhesion and wound recovery, such as the Rac signaling and actin-nucleation by ARP-WASP complex pathway, were upregulated on 800 nm and 1000 nm topography. In our wound recovery assay, we observed that filopodia in the leading edge cell membrane emerged as elongated finger-like cellular protrusion that aligned with the underlying 800 nm and 1000 nm ridge width patterns, while it was fan-like morphology on flat silk films.

When we targeted Cdc42 with the specific inhibitor ML141, we observed delayed wound recovery with inhibition of filopodia length, less alignment to patterns, and lower filopodia density. Although the long population of filopodia decreased after ML141 treatment on 1000 and 800 nm patterned silk films, the wound recovery rate did not significant decrease. This could be related to the amount of long filopodia still remaining after ML141 inhibition, which still could be sufficient for promoting cell migration and wound recovery. When ML141 was administered in vivo, we found a profound delay on corneal wound healing and full re-epithelization of the injured cornea occurred after 3.5 days post final addition of ML141 as eyedrops. Taken together, our data showed that nanotopography in combination with collagen I increased cell number and wound recovery through a mechanism related to FA and integrin signaling that induce filopodia formation, as a way to sense the environment, and enhance cellular migration when the appropriate signals are in place.

## Conclusion

Collagen I and nanotopography improved the cell adherence to silk films, and helped cells form better attachments that resulted in improved wound healing. Collagen alone is difficult to pattern into topography and may be of limited clinical use, but collagen in combination with patterned silk films may provide better in vivo wound healing and improve the use of silk films for corneal transplantation therapies.

## Supplementary information

Supplementary Video 1.

Supplementary Video 2.

Supplementary Information 1.
