# Supplementary material for: Silk films with nanotopography and extracellular proteins enhance corneal epithelial wound healing
Source: Sci Rep. 2021 Apr 14;11:8168. doi: 10.1038/s41598-021-87658-1 (PMC8046786; doi:10.1038/s41598-021-87658-1)
Supplement: Supplementary file 3 — Supplementary Information 1. [file 41598_2021_87658_MOESM3_ESM.docx]

Title: Silk films with nanotopography and extracellular proteins enhance corneal epithelial wound healing.

Supplementary Materials

Figures S1-S4


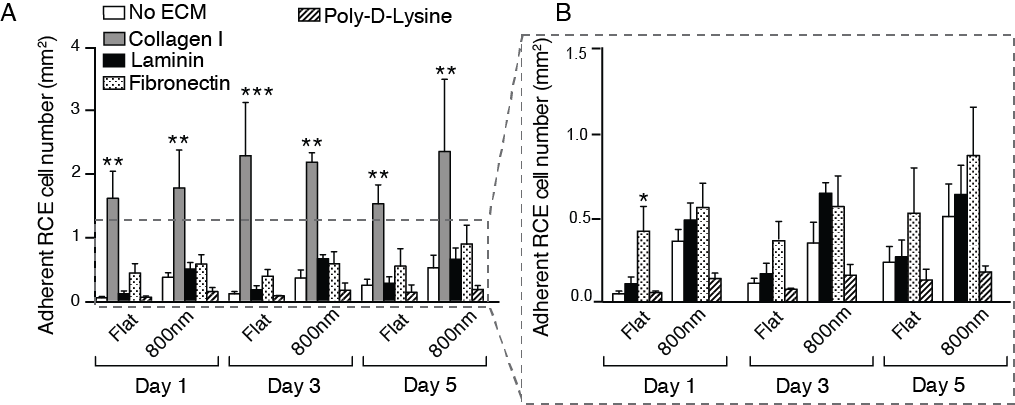


**Figure S1.** Collagen I coating of silk films enhanced cellular adhesion and growth of Rabbit corneal epithelial cells (RCEC). RCEC were seeded on uncoated or collagen I, laminin, fibronectin or Poly-D-Lysine coated flat or 800 nm ridge width silk topographies. (A) Quantification of cell number shows that 5 fold more adherent cells were present on collagen I coated silk in comparison to other ECMs. (B) Detailed analysis of other ECMs, showed higher cell number on fibronectin coated films when compared to laminin or Poly-D-Lysine. In general, cell number increased on 800 nm patterned silk film in comparison to flat silk film. (* indicate p<0.05, ** indicate p<0.005, ***indicate p<0.0005 when compared between collagen I and other ECMs at one surface, values are means± SEM).


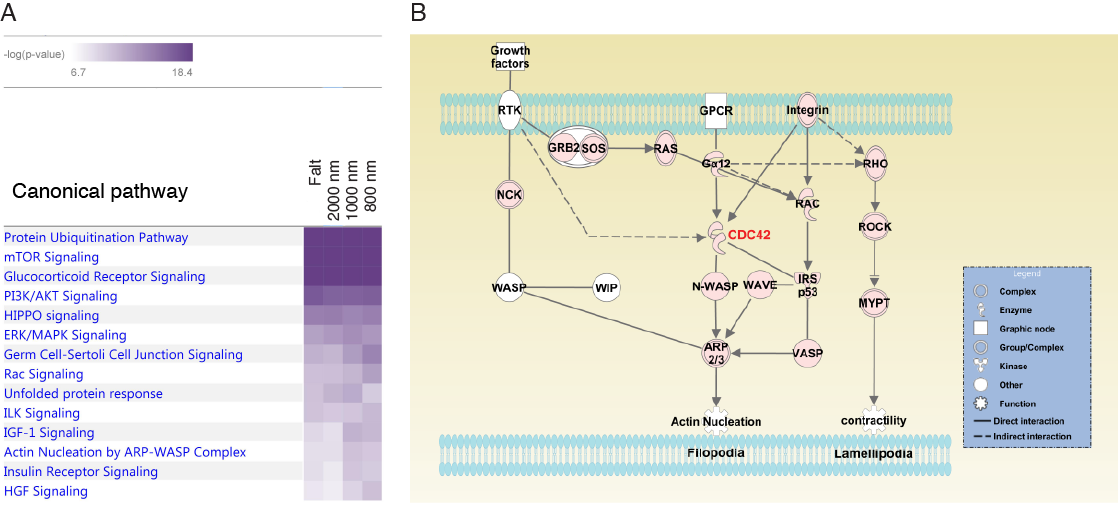


**Figure S2.** Ingenuity pathway analysis (IPA) showed that several canonical pathways were upregulated by the nano-topography on silk film. (A) IPA differentially expressed genes for HCEC cultured on each patterned silk substrate in comparison to cultures on flat silk film for 3 days. IPA analysis found the top signaling pathways involved and the corresponding p values. (B) The Actin nucleation by ARP-WASP complex pathway was selected as a candidate for their role on cell adhesion, migration and proliferation and because it becomes activated by mechanotransduction signaling. Genes in pink color indicated differentially upregulated genes on 800nm patterned topography when compared to flat silk film. This pathway indicates that the signal start when the cells sense biophysical cues or ECM ligand provided by nano-topography on silk or collagen I through integrin receptors and then leads to integrin receptor aggregation and recruitment and clustering of Rho family of small GTPases, which included Rho and Rac. Then it initiates a cascade of signaling events that globally regulate dynamic actin rearrangements. The signaling cascade included activation of ROCK and MYPT leading to the formation of contractile actin filaments and formation of lamellipodia. A parallel pathway may start from integrin-binding and activation of Cdc42, which can also be induced by Rac signaling. These two pathways activate the ARP2/3 complex which induces actin nucleation and the formation of filopodia.

**Figure S3.** Aligned and extended filopodia were formed mainly on the 1000 nm and 800 nm ridge width patterned silk films. RCEC were seeded on silk film with different topographies and let grown until confluency. Then a scratch assay was performed and time lapse images were obtained to determine the wound recovery rate. Phase-contrast images shown after 7 hours post injury demonstrate that filopodia formation occurs in all topographies, but it only aligned and extended on1000 nm and 800 nm patterned silk films. Nano-topography influenced filopodia transforming the smooth fan-shape morphology on flat to finger-like protrusion on patterned silk film substrate.

**Figure S4**. ML141 delays in vivo corneal epithelial wound healing. Wild-type C57BL/6 mice were subjected to 2-mm central corneal epithelial debridement on the right eye only and immediately injected subconjunctivally with 250µM of ML141 or received 15 µl eyedrops of 500µM ML141. A control group received vehicle as eyedrops of equal volume. Fluorescein staining was used to follow the re-epithelialization of the injured corneas using a slit lamp biomicroscope. (A) In animals receiving ML141, we observed a persistent delay in epithelial wound closure compared with control mice. The re-epithelialization was strongly delayed in the mice that received ML141 eyedrops, and in this group complete healing was observed only 3.5 days after the final eyedrop dose given after 32 h post wounding. (B) Quantification of the images confirms that epithelial wound closure is delayed upon ML141 treatment. In control mice the epithelium was almost healed at 30 h but only 75% healed in mice that received subconjunctival injection of ML141, while 0% healed in the group that received ML141 as eyedrops. Data are expressed as mean ± SEM (n = 3/group). *P ≤ 0.01.

**Author details**

First author:

Yuncin Luo

Email address: [yunchinglo@icloud.com](mailto:yunchinglo@icloud.com)

Primary affiliation: University of Illinois at Chicago, United States

Funders:

National institutes of Health, K12EY021475

National institutes of Health, R21EY019561

National institutes of Health, P30EY001792

Research to Prevent Blindness Career Development Award

Falk Medical Research Trust Catalyst Awards Program

Second author:

Kai B. Kang

Email address: [kaikang@uic.edu](mailto:kaikang@uic.edu)

Primary affiliation: University of Illinois at Chicago, United States

Funders:

National institutes of Health, K12EY021475

National institutes of Health, R21EY019561

National institutes of Health, P30EY001792

Research to Prevent Blindness Career Development Award

Falk Medical Research Trust Catalyst Awards Program

Third author:

Rachel Sartaj

Email address: [rachelsartaj@gmail.com](mailto:rachelsartaj@gmail.com)

Funders:

Primary affiliation: University of Illinois at Chicago, United States

National institutes of Health, K12EY021475

National institutes of Health, R21EY019561

National institutes of Health, P30EY001792

Research to Prevent Blindness Career Development Award

Falk Medical Research Trust Catalyst Awards Program

Fourth author

Michael Sun

Email address: [msun8@uic.edu](mailto:msun8@uic.edu)
Primary affiliation: University of Illinois at Chicago, United States

Funders:

National institutes of Health, K12EY021475

National institutes of Health, R21EY019561

National institutes of Health, P30EY001792

Research to Prevent Blindness Career Development Award

Falk Medical Research Trust Catalyst Awards Program

Fifth author

Qiang Zhou

Email address: [jackzhou@uic.edu](mailto:jackzhou@uic.edu)

Primary affiliation: University of Illinois at Chicago, United States

National institutes of Health, K12EY021475

National institutes of Health, R21EY019561

National institutes of Health, P30EY001792

Research to Prevent Blindness Career Development Award

Falk Medical Research Trust Catalyst Awards Program

Sixth author

Victor Guaiquil

Email address: [vguaiqui@uic.edu](mailto:vguaiqui@uic.edu)

Primary affiliation: University of Illinois at Chicago, United States

National institutes of Health, K12EY021475

National institutes of Health, R21EY019561

National institutes of Health, P30EY001792

Research to Prevent Blindness Career Development Award

Falk Medical Research Trust Catalyst Awards Program

Corresponding author:

Mark I. Rosenblatt

Email address: [mrosenbl@uic.edu](mailto:mrosenbl@uic.edu)

Primary affiliation: University of Illinois at Chicago, United States

National institutes of Health, K12EY021475

National institutes of Health, R21EY019561

National institutes of Health, P30EY001792

Research to Prevent Blindness Career Development Award

Falk Medical Research Trust Catalyst Awards Program

**Author contributions**

Y.L. conceived the project, designed the experimental approach, performed experiments, analyzed the data and interpretation, manuscript writing, final approval of manuscript, and administrative support.

K.K. conceived the concept of gene analysis and performed experiments to determine the IPA.

R.S. conceived the concept of coating protein and developed primary mouse corneal culture.

M.S. conceived the project and performed experiments to provide silk films.

Q.Z. conceived the concept of primary corneal culture and performed in vivo experiments.

V.G. conceived the project, designed the experimental approach, supervised the project, data analysis and interpretation, manuscript writing, final approval of manuscript, and administrative support.

M.R. conceived the project, designed the experimental approach, supervised the project, financial support, data analysis and interpretation, manuscript writing, final approval of manuscript, and administrative support.
